# LIA in arthroplasty – the history of a single-center observational study leading to implementation in general clinical practice

**DOI:** 10.1080/17453674.2020.1763563

**Published:** 2020-05-14

**Authors:** Henrik Kehlet

**Affiliations:** Rigshospitalet, Section of Surgical Pathophysiology, Copenhagen, Denmark

Although local anesthetic wound infiltration has been widely used for more than 50 years in different surgical procedures, the observational study by Kerr and Kohan (2008) in 325 patients undergoing 3 different hip and knee arthroplasties led to an increased attention to the very simple, surgeon-administrated technique of high-volume local anesthetic wound infiltration (LIA), with claimed effects on pain, mobilization, opioid use and hospital stay. This study was a non-RCT performed by 2 physicians in a small private hospital in Sydney, factors that usually argue against the value of such reports. Nevertheless, in this case the article further stimulated others to do original and more scientific research including RCT’s and later followed by several systematic reviews. However, these reviews unfortunately often suffered from methodological problems, since the efficacy of LIA (with NSAID) was not compared to appropriate control groups including systemic NSAIDs and paracetamol (Andersen et al. 2014). Also, although the original suggestion that ketorolac and epinephrine should be added in the solution in some way was rational, it was not proven in later studies to be necessary or essential compared to systemic NSAIDs/Cox 2 inhibitors (Spreng et al. 2010, Schotanus et al. 2017).

Whatsoever, history has shown that the observational study by Kerr and Kohan (2008) stimulated much research to argue that the technique is simple, safe and effective in TKA, but apparently not in THA (Andersen et al. 2014, Tan et al. 2019, Wainwright et al. 2020). The reasons for this discrepancy between TKA and THA still needs to be clarified but may be related to wound size and the fact that pain is less following a THA compared with TKA.

Concomitant with the availability of more scientific data on LIA, the concept of fast-track THA and TKA was undergoing much progress. Since optimal patient management is a prerequisite for enhanced recovery and early mobilization, LIA most probably may have played an important role in this process, although a direct association is difficult to prove.

The suggestion that further efficacy can be obtained by a wound catheter is controversial (Andersen et al. 2014, Wainwright et al. 2020) although this author personally observed an effect in a patient the day after a knee replacement in the orthopedic hospital in Sydney when visiting Kerr and Kohan.

Although safe, simple and effective, however, there is still a demand to take the time to do the infiltration meticulously to cover all important wound layers and probably also to do further studies on which tissue layers and places to be most important to infiltrate to obtain sufficient analgesia (Andersen et al. 2014). In this context, more information is needed on the additional analgesic effect of performing a surgeon-administered “blind” saphenous block when being inside the joint. Finally, although many studies are available, we need more data from comparing or combining LIA with different peripheral nerve blocks (Zhang et al. 2018, Sardana et al. 2019).

In conclusion, this study is a nice example of an idea that subsequently has been well-documented to be useful and furthermore implemented in clinical practice in many centers around the world as well as in clinical guidelines.(Soffin et al. 2019a and b, Wainwright et al. 2020).

Henrik Kehlet *Rigshospitalet, Section of Surgical Pathophysiology,* *Copenhagen, Denmark* *E-mail:*henrik.kehlet@regionh.dk
